# miR-335-3p improves type II diabetes mellitus by IGF-1 regulating macrophage polarization

**DOI:** 10.1515/med-2024-0912

**Published:** 2024-02-29

**Authors:** Zhengzheng Ju, Fan Cui, Zheng Mao, Zhen Li, Xiayu Yi, Jingjing Zhou, Jinjin Cao, Xiaoqin Li, Zengkun Qian

**Affiliations:** Department of Clinical Laboratory, Wuhu Hospital Affiliated to Anhui University of Science and Technology (The First People’s Hospital of Wuhu), Wuhu, Anhui, China

**Keywords:** type II diabetes mellitus, miR-335-3p, IGF-1, macrophages polarization

## Abstract

Previous studies have found that miR-335 is highly expressed in type II diabetes mellitus (T2DM) models and is related to insulin secretion, but there are few studies on the regulatory effects of miR-335-3p on insulin resistance and macrophage polarization in T2DM patients. This study aims to explore the effects of miR-335-3p on insulin resistance and macrophage polarization in T2DM patients. Blood glucose (insulin tolerance tests, glucose tolerance tests) and body weight of the T2DM model were measured; macrophages from adipose tissue were isolated and cultured, and the number of macrophages was detected by F4/80 immunofluorescence assay; the Real-time quantitative polymerase chain reaction (qPCR) assay and Western blot assay were used to detect the miR-335-3p expression levels, insulin-like growth factor 1 (IGF-1), M1-polarizing genes (inducible nitric oxide synthase [iNOS] and TNF-α) as well as M2-polarizing genes (IL-10 and ARG-1). The targeting link between miR-335-3p and IGF-1 was confirmed using bioinformatics and dual luciferase assay. The results showed that miR-335-3p expression level in adipose tissue of the T2DM model was significantly decreased, and the mice’s body weight and blood glucose levels dropped considerably, miR-335-3p inhibited the number of macrophages, inhibiting the iNOS and TNF-α relative mRNA expression levels, and up-regulated the IL-10 and ARG-1 relative mRNA expression levels, miR-335-3p negatively regulated target gene IGF-1, IGF-1 significantly increased the iNOS and TNF-α mRNA and protein expression levels, decreasing the IL-10 and ARG-1 mRNA and protein expression levels, indicating that miR-335-3p could affect the T2DM process by regulating macrophage polarization via IGF-1.

## 1 Introduction

Type II diabetes mellitus (T2DM), the quantity of patients with which has been increasing worldwide, seriously affects their quality of life. Effective prevention and treatment methods for T2DM have been urgently needed [1]. The development of T2DM is significantly influenced by chronic inflammation of adipose tissue [2,3,4]. The adipose tissue’s chronically low inflammation manifests chronically high levels of adipose tissue inflammatory molecules, large numbers of adipose tissue macrophage infiltration, and continuous activation of inflammatory cell signaling [5].

Macrophages in adipose tissue are the main effector cells that trigger T2DM-related inflammation [6]. Macrophages are classified into two types: pro-inflammatory (M1) and anti-inflammatory (M2). Among them, the M1-type macrophage can secrete pro-inflammatory cytokines and induce the onset of insulin resistance as well as inflammation in adipose tissue. On the contrary, M2-type macrophages are capable of secreting cytokines that inhibit inflammation and have an anti-inflammatory effect [7,8].

Insulin-like growth factor 1 (IGF-1), named for its structural similarity to insulin, can increase insulin sensitivity and improve insulin function [9,10]. Also, IGF-1 improves T2DM physiological function by binding to IGF-1R [11]. It has been shown that miR-190b can directly target IGF-1 and thus affect lipid metabolism and insulin signaling [12].

microRNA (miRNA) belongs to single-stranded non-coding small molecule RNA. The length is about 22 nucleotide sequences. It controls a variety of biological processes, such as metabolism, cell differentiation, apoptosis, and cell proliferation. Also, it is connected to the occurrence and progression of various diseases [13].

Recent studies have demonstrated that miRNAs could also contribute to insulin resistance, the onset and development of diabetes, and its complications [14]. In addition, miRNAs can significantly affect the treatment of inflammation-related diseases by regulating macrophage polarization [15,16,17,18]. miR-335 connects inflammation with poor adipose tissue metabolism because it is involved in lipid activity metabolism and adipocyte differentiation [19]. Numerous studies have shown that miR-335 is substantially expressed in T2DM models and connected to insulin secretion [20,21]. Related studies have confirmed that miR-335-3p can be aberrantly expressed in a mouse model which is induced by a high-fat diet, affecting the development of insulin resistance and glucolipid metabolism [22,23].

However, there are fewer studies on the regulatory impact of miR-335-3p on insulin resistance as well as macrophage polarization in T2DM. Therefore, this study primarily aims to explore the miR-335-3p effects on insulin resistance as well as the T2DM macrophage polarization, studying the in-depth mechanism. This will lay a theoretical foundation for revealing the miRNA action mechanism in the development of diseases such as T2DM, as well as provide clues for the development of drugs using miRNAs as therapeutic targets.

## 2 Materials and methods

### 2.1 Animals

Hunan SJA Laboratory Animal Co., LTD provided thirty 8-week-old SPF-grade C57BL/6 mice (12–25 g). The experiment will be conducted strictly according to international practice following the statement issued by the Association for Research in Vision and Ophthalmology. All animal experiments complied with the ARRIVE guidelines. The experimental protocol was approved by the Animal Ethics Committee of Wuhu Hospital Affiliated to Anhui University of Science and Technology.

### 2.2 Mice modeling

A regular diet (15% lipids/kcal) and a high-fat diet (HFD, 60% lipids/kcal), each fed to 30 8-week-old SPF-grade C57BL/6 mice, were classified into two groups of 15 animals each at random. Mice received 3.5 ± 0.3 g of food per day. Body weight was assessed 8 weeks later. To identify the T2DM mice model, insulin tolerance tests (ITT) and glucose tolerance tests (GTT) were performed.

### 2.3 GTT

Mice were starved for an entire night before the experiment. The mice were gavaged with 2 g/kg of a 20% glucose saline solution, and at 0 min, blood was drawn from the tail and the blood glucose level was determined. Then, blood was taken at 15, 30, 60, 90, and 120 min to measure the blood glucose [24].

### 2.4 ITT

Before the experiment, mice fasted overnight and 0.5 IU insulin/kg/bw was injected intraperitoneally. Then, blood was extracted from the tail vein at intervals of 0, 30, 60, 90, and 120 min for the detection of blood glucose [24].

### 2.5 Isolation and culture of macrophages in adipose tissues

Mice were euthanized using the carbon dioxide inhalation method, and epididymal adipose tissue was isolated. Digestion of epididymal adipose tissue in collagenase solution (100 mg/mL in Krebs–Henseleit buffer) was performed at 37°C for 45 min with rocking (100 times/min). Cells were filtered through a nylon mesh (100 µm) and washed three times with Krebs Henseleit buffer. At 4°C in 500 × *g* centrifugation for 10 min to separate adipocytes and macrophages. Macrophages (CD11b + MicroBeads) were purified by magnetically activated cell sorting (Miltenyi Biotec, Germany), and macrophages were identified by flow cytometry with purity >95% [25]. Following isolation, the macrophages were cultured at 37℃ in a 5% CO_2_ incubator in RPMI-1640 media with 10% fetal bovine serum.

In addition, M1/M2 polarization of macrophages was induced respectively with lipopolysaccharide (LPS) and IL-4. After 6 h of macrophage starvation, macrophages were incubated respectively with 10 ng/mL IL-4 and 1 μg/mL LPS for 24 h, for the induction of macrophage M1/M2 polarization.

### 2.6 Adenovirus injection

To analyze the miR-335-3p impact on T2DM model mice, the mice were fixed first. Take a fixed cylinder with a blind end at one end, and the diameter of the cylinder exactly accommodates a mouse. The cylinder has multiple ventilation holes, and use the mouse’s habit of drilling holes to let it enter the cylinder. There is a circular hole on the back cover of the cylinder, which exactly accommodates the tail. Put the mouse tail through the hole and cover the back cover. Then, the tails of the mice were rubbed with 75% alcohol cotton balls, and finally, 100 μL of adenovirus-coated Ad-miR-335-3p (10 mg/mL) was injected into the same side tail vein every 56 h, 3 times a week for 3 weeks. Besides, body weight and blood glucose levels were measured after injection, and blood glucose levels were measured every half hour for the first 2 h to observe the dynamic changes of blood glucose.

### 2.7 Real-time quantitative polymerase chain reaction (real-time qPCR)

The relative mRNA expression levels of miR-335-3p, inducible nitric oxide synthase (iNOS), TNF-α, IL-10, Arg-1, and IGF-1 were assessed in each group of experiments using RT-qPCR. The whole RNA was extracted using the TRIzol method. The cDNA was reverse-transcribed using the Revert Aid First Strand cDNA Synthesis kit. An AB 7500 Real-Time PCR System and an SYBR-Green PCR Master Mix kit were used to perform RT-qPCR. TAKRA contributed U6, miR-335-3p, iNOS, TNF, IL-10, Arg-1, and primers for GYS1 and GAPDH. The primer sequences are listed in Table 1.

**Table 1:** Primer sequences

	Forward primer	Reverse primer
U6	5′-AGGATGTAGTCGTGGAACTC-3′	5′-GTTTGATATTGAGGTACTGGTCC-3′
miR-335-3p	5′-GGGTCAAGAGCAATAACGA-3′	5′-GGCTATAACCCATGAGAGGG-3′
iNOS	5′-CCAACAATACAAGATGACCCT-3′	5′-TTCTGGAACATTCTGTGCTG-3′
TNF	5′-TTCTCATTCCTGCTTGTGG-3′	5′-TTGGGAACTTCTCATCCCT-3′
IL-10	5′-GCATGTTGCCTGCTCTT-3′	5′-ATGCTCCTTGATTTCTG-3′
Arg-1	5′-GAACTGAAAGGAAAGTTCCCA-3′	5′-GAACTGAAAGGAAAGTTCCCA-3′
GYS1	5′-CATCTCCACCAACCTCTCC-3′	5′-AGAATGTAAATGCCGTAAGCTG-3′
GAPDH	5′-ACTCTTCCACCTTCGATGC-3′	5′-CCGTATTCATTGTCATACCAGG-3′

### 2.8 F4/80 immunofluorescence staining

F4/80 immunofluorescence staining was performed for the identification of the mouse adipose tissue macrophages of the miR-335-3p overexpression transfected model. Tissue sections were boiled for 10 min in a pot containing 10 mol/L sodium citrate (pH 6.0). Mouse anti-F4/80 antibody (1:500) was added to phosphate-buffered saline (PBS) containing 2% bovine serum albumin and 0.1% Triton X100, and the mixture was then incubated overnight at 4℃. PBS was washed three times. Then Alexa594-conjugated anti-rabbit antibody was added, being incubated overnight at 4℃. Next, PBS was stained with 4′,6-diamidino-2-phenylindole after being rinsed three times. Each area’s total quantity of F4/80 positive cells as well as F4/80 positive cells with nuclei were calculated. The percentage of F4/80 positive cells was calculated as a result.

### 2.9 Dual luciferase report assay

In 24-well plates, cells were plated and cultivated until cell fusion reached 70%. Through the use of Lipofectamine 2000 transfection reagent, the cells were simultaneously transfected with the luciferase reporter plasmid (0.5 g), NC mimics, as well as miR-335-3p mimics (100 nmol/L). Using the proper Luciferase Reporter Gene Assay Kit technique, luciferase activity was evaluated after 48 h.

### 2.10 Western blot (WB)

The WB assay was performed to explore the expression levels of IGF-1, iNOS, TNF-α, IL-10, and Arg-1 proteins. A bicinchoninic acid protein assay kit was used to extract the RIPA lysate’s total protein content and measure it. sodium dodecyl sulphate-polyacrylamide gel electrophoresis was used to electrophoretically separate protein samples before transferring them to PVDF membranes. One hour of incubation was conducted using a blocking buffer containing 5% skim milk powder. After washing, rabbit anti-IGF-1 (1:1,000 dilution), iNOS (1:1,000 dilution), TNF-α (1:500 dilution), IL-10 (1:1,000 dilution), and Arg-1 (1:1,000 dilution) polyclonal antibodies (primary antibody) were added to incubate overnight at 4℃. Wash three times for 10 min each time, and the corresponding HRP-labeled secondary antibody (1:1,500 dilution) was added to incubate for 2 h at room temperature. The Esports Champion League method was applied to develop with β-actin as a reference.

### 2.11 Macrophage transfection

Macrophage transfection was performed by Lipofectamine 2000 transfection reagent. And cells were inserted into six-well plates (1 × 10^4^ cells/well), being cultured for 12 h. Next, cells were transfected by pc-NC, pc-IGF-1, IL4 + NC mimic + pc-NC, IL4 + miR-335-3p mimic + pc-NC, IL4 + miR-335-3p mimic + pc-IGF-1, LPS + NC mimic + pc-NC, LPS + miR-335-3p mimic + pc-NC, and LPS + miR-335-3p mimic + pc-IGF-1 vectors. Cells were incubated in six-well plates for 48 h. Then, they were collected for subsequent analysis. The concentration of miR-335-3p mimic was 50 nmol/L.

### 2.12 Statistical methods

Three separate independent runs of each experimental group were conducted. Analysis of variance and *t* tests were used to compare groups and SPSS 18.0 was utilized for statistical analysis. The outcomes of the data are shown as mean ± standard deviation. When *P* < 0. 05 was reached, the difference was deemed statistically significant.

## 3 Results

### 3.1 Mouse T2DM model construction

**Figure 1:**
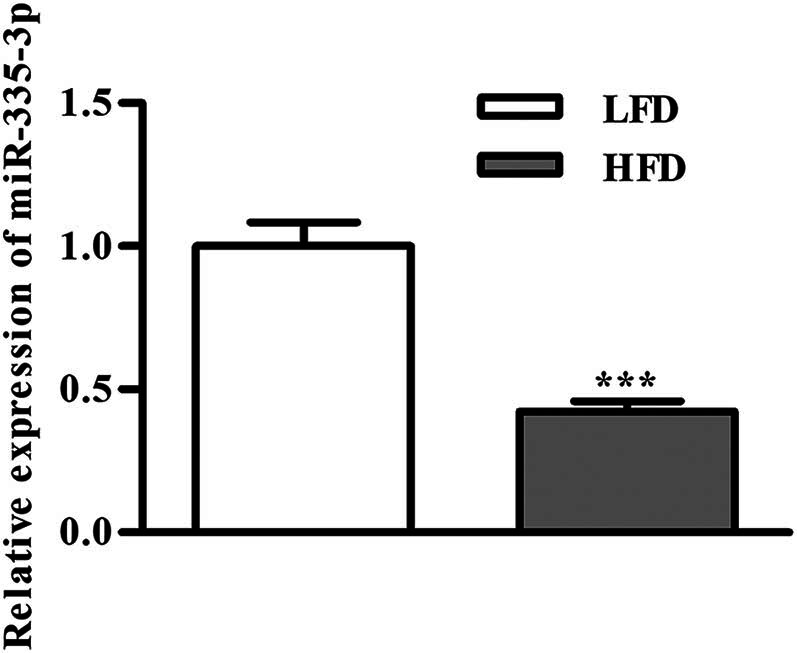
The expression level of miR-335-3p in mouse T2DM model. ^***^ *P* < 0.001 is the comparison with the LFD group.

**Figure A1:**
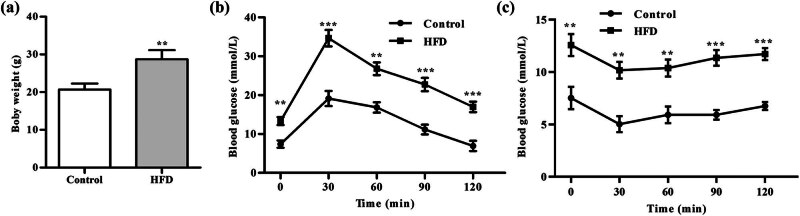
Body weight and blood glucose detection of mouse T2DM model. A is the result of body weight; B. Plasma glucose levels during the glucose tolerance test (GTT); C. Plasma glucose levels during the insulin tolerance test (ITT). ** *P* <0.01, *** *P* < 0.001 is the comparison with the Control group.

As shown in Figure 1a, in the HFD group, the mice’s body weight (Figure A1a) was greatly higher in comparison with the control group. Also, the results of the GTT and ITT experiments showed that the blood glucose (Figure A1b and c) levels of mice were greatly higher in the HFD group than in comparison to the control group. Thus, the results indicated that the T2DM mouse model was modeled and showed insulin resistance.

### 3.2 Expression of miR-335-3p in adipose tissue of mouse T2DM model

Epididymal adipose tissues from the T2DM mice were used to separate macrophages. RT-qPCR was used to analyze the level miR-335-3p mRNA’s relative expression. As shown in Figure 1, when in comparison with the LFD group, the HFD group’s miR-335-3p mRNA expression level in macrophages was considerably lower.

### 3.3 Role of miR-335-3p high expression on the model

**Figure 2:**
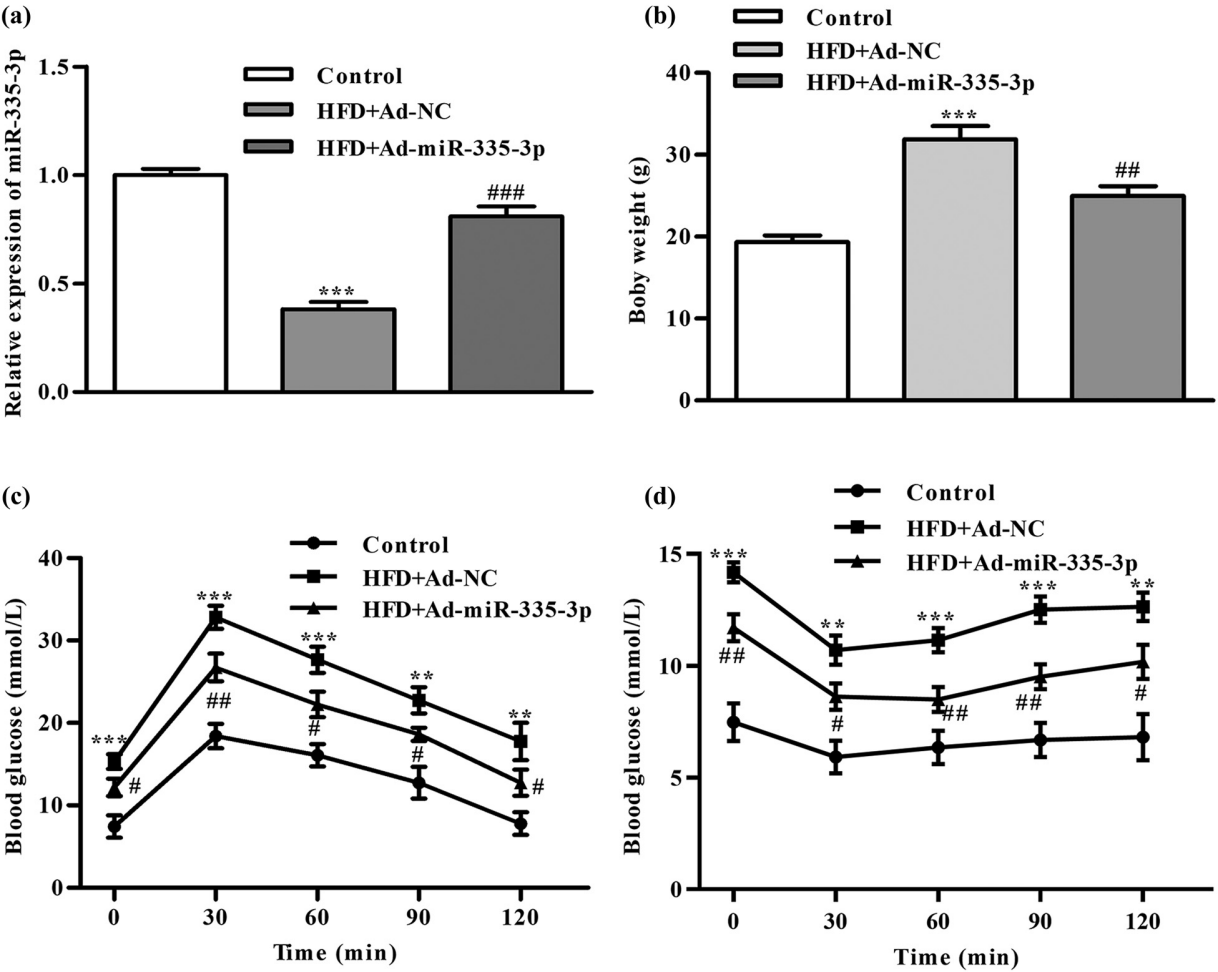
Effect of miR-335-3p overexpression on mouse T2DM model. (a) The miR-335-3p expression level. (b) The result of weight measurement. (c and d) The results of blood glucose measurement by GTT and ITT assays. ^**^ *P* < 0.01, ^***^ *P* < 0.001 is the comparison with the control group, ^#^ *P* < 0.05, ^##^ *P* < 0.01, ^###^ *P* < 0.001 is the comparison with HFD + Ad-NC group.

To explore the impact of miR-335-3p high expression on the model, adenovirus-encapsulated miR-335-3p was injected intraperitoneally into the mouse model. The findings showed that the expression levels of mRNA in miR-335-3p got greater in macrophages in the HFD + Ad-miR-335-3p group in comparison with the HFD + Ad-NC group (Figure 2a). The findings showed that in the HFD + Ad-miR-335-3p group, mice exhibited considerably lower body weight in comparison with the HFD + Ad-NC group (Figure 2b). However, the findings from the GTT experiments demonstrated that mice overexpressing miR-335-3p exhibited reduced blood glucose levels when compared to the HFD + Ad-NC control group (Figure 2c). ITT revealed that overexpression of miR-335-3p reduced blood glucose levels after insulin injection (Figure 2d). These findings indicated that overexpression of miR-335-3p relieves insulin resistance.

### 3.4 Detection of macrophage polarization in mouse adipose tissue

**Figure 3:**
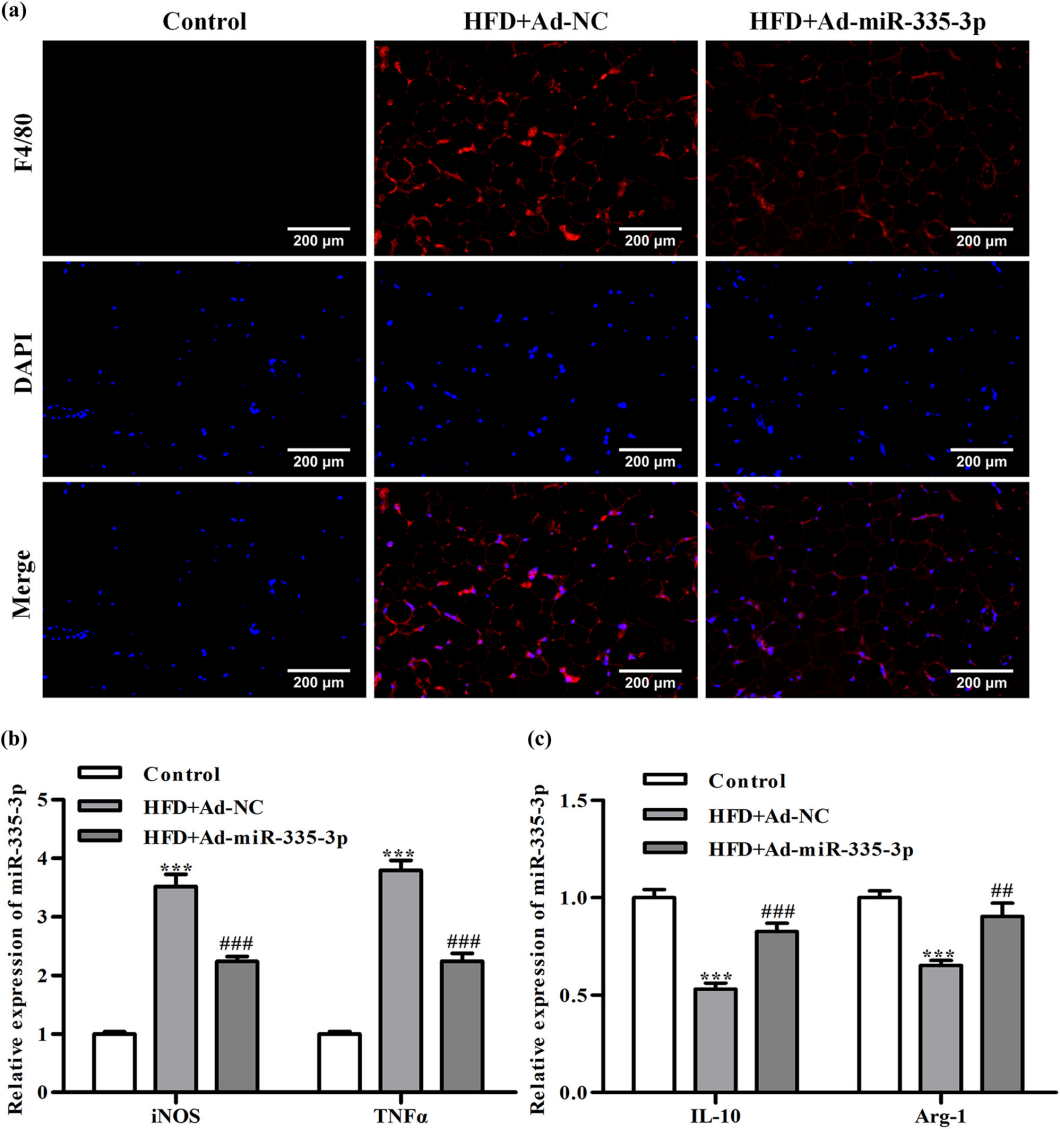
The detection of macrophage polarization in mouse tissue. (a) The result of immunofluorescence detection of F4/80; (b) the detection of iNOS and TNF-α mRNA levels; and (c) the detection of IL-10 as well as Arg-1 mRNA level. ^***^ *P* < 0.001 is the comparison with the control group, ^##^ *P* < 0.01, ^###^ *P* < 0.001 is the comparison with the HFD + Ad + NC group.

According to Figure 3a, F4/80 immunofluorescence detection revealed that mice in the HFD + Ad-NC group had considerably more F4/80 positive cells in their adipose tissue than mice in the control group. It showed that the adipose tissue’s macrophage population had risen as a result of HFD. In addition, when compared to the HFD + Ad-NC group, miR-335-3p dramatically reduced the quantity of F4/80 positive cells in the adipose tissue of mice (Figure 3a).

RT-qPCR was performed to detect macrophage M1/M2 polarization marker genes. The results showed that the HFD + Ad-NC group had higher expression levels of the M1-type macrophage pro-inflammatory marker genes (iNOS and TNF-α) than did the control group. In addition, in comparison to the HFD + Ad-NC group, the expression levels of iNOS and TNF-α were lower in the HFD + Ad-miR-335-3p group (Figure 3b). Anti-inflammatory gene expression in M2-type macrophages (IL-10 and Agr-1) was lower in the HFD + Ad-NC group than it was in the control group. However, the expression of the genes for IL-10 and Agr-1 was dramatically elevated by miR-335-3p (Figure 3c). This demonstrated that miR-335-3p could instruct HFD mouse macrophages to retrain their M1 polarization.

### 3.5 Relationship between miR-335-3p and IGF-1 targeting

**Figure 4:**
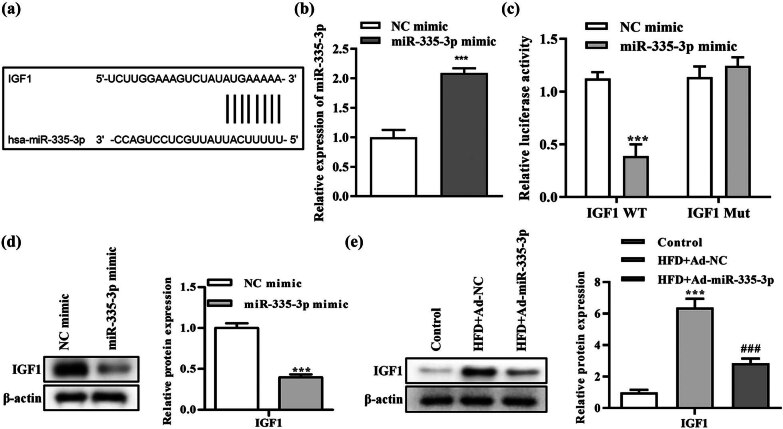
The targeting relationship between miR-335-3p and IGF-1. (a) A bioinformatics analysis of miR-335-3p combining site to IGF-1 and (b) the efficiency of RT-qPCR for transfection of miR-335-3p mimic. (c) A quantitative analysis of dual luciferase assay and (d) Western blot detection of GYS1 protein expression level. ^***^ *P* < 0.001 is the comparison with NC mimic group; (e) RT-qPCR detection of IGF-1 mRNA expression. ^**^ *P* < 0.01 is the comparison with the control group.

IGF-1 was the target gene of miR-335-3p, according to bioinformatics analysis, and it could bind to the 3UTR of IGF-1 Wt (Figure 4a). RT-qPCR showed that the expression of miR-335-3p was significantly increased after transfection of miR-335-3p mimic (Figure 4b). The findings of the dual luciferase reporter gene assay showed that the miR-335-3p mimic group’s IGF-1 WT had significantly lower luciferase activity than the control NC mimic group, while no significant change was found in IGF-1 Mut (Figure 4c). When compared with the NC mimic group, the IGF-1 protein expression level was considerably lower than that in the miR-335-3p mimic group, as shown in Figure 4d. Comparing the HFD + Ad-N group to the control group, the level of IGF-1 protein expression was significantly higher in the HFD + Ad-N group. In addition, miR-335-3p markedly reduced IGF-1 protein expression (Figure 4e). This suggested that miR-335-3p adversely regulated the target gene IGF-1 in the T2DM mouse model.

### 3.6 Effect of IGF-1 on macrophage polarization

**Figure 5:**
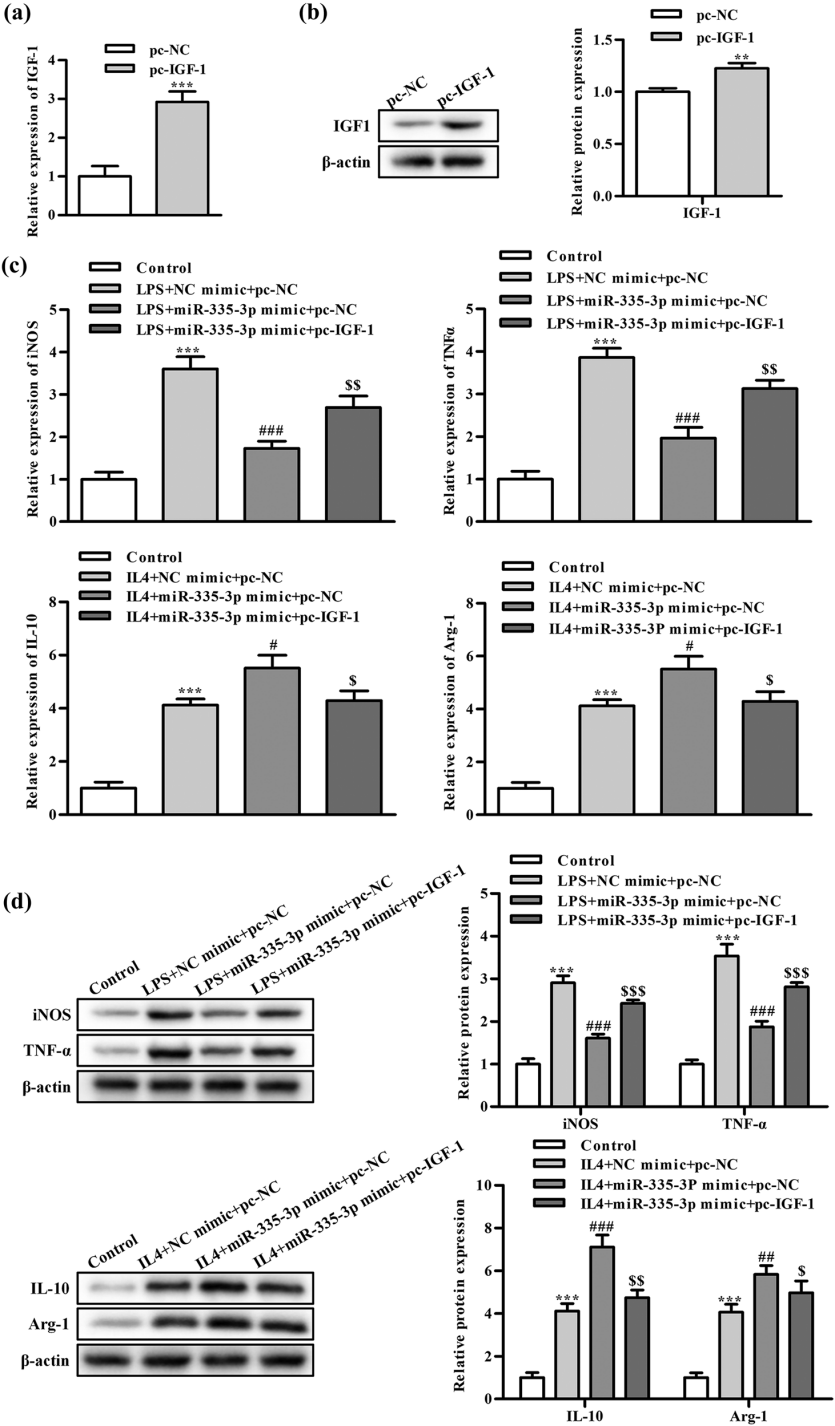
Impact of IGF-1 on the polarization of macrophages. (a) The RT-qPCR detection of IGF-1 mRNA expression level. ^***^ *P* < is the comparison with the pc-NC group; (b) WB detection of GYS1 protein expression level; (c) RT-qPCR detection of polarized gene mRNA expression level; and (d) WB detection of polarized gene protein expression level. ^**^ *P* < 0.01, ^***^ *P* < 0.001 is the comparison with the control group. ^##^ *P* < 0.01, ^###^ *P* < 0.001 is the comparison with LPS/IL-4 + NC mimic + pc-NC group, ^$^ *P* < 0.05, ^$$^ *P* < 0.01 is the comparison with LPS/IL-4 + miR-335-3p mimic + pc-NC group.

We transfected IGF-1 into macrophages to further confirm the role of IGF-1 in the M1/M2 polarization of macrophages implicated in miR-335-3p. As can be shown in Figure 5a and b, RT-qPCR and WB results revealed that transfected macrophages had considerably higher levels of IGF-1 mRNA and protein expression compared to the pc-NC group. It indicated that IGF-1 transfected macrophages with better efficiency.

In addition, macrophages were stimulated through LPS and IL-4 and transfected through IGF-1, respectively. RT-qPCR assay and WB assay were applied to identify mRNA and protein expression levels of M1 or M2 polarization markers in macrophages. Experiments were divided into the control group, LPS/IL-4 + NC mimic + pc-NC group, LPS/IL-4 + miR-335-3p mimic + pc-NC group, and LPS/IL-4 + miR-335-3p mimic + pc-IGF-1 group. As shown in Figure 5c and d, as the comparison with the control group, iNOS and TNF-α were greatly higher in levels of expression in both mRNA and protein in the LPS + NC mimic + pc-NC group. IL-10 and Agr-1 were significantly higher in expression levels of both mRNA and protein in the IL-4 + NC mimic + pc-NC group. It indicated that LPS and IL-4 successfully converted macrophages to M1/M2 type, respectively. The miR-335-3p mimic significantly downregulated iNOS, TNF-α mRNA, and protein expression levels, compared with LPS/IL-4 + NC mimic + pc-NC group, and significantly increased IL-10, Agr-1 mRNA and protein expression levels, compared with LPS/IL-4 + miR-335-3p mimic + pc-NC group. IGF-1 markedly changed the expression levels of IL-10, TNF-α, iNOS, and Agr-1 proteins as well as their mRNA and protein counterparts. This suggested that IGF-1 prevented IL-4-induced macrophages from converting to M2-type anti-inflammatory-acting macrophages, favoring the conversion of LPS-induced macrophages to M1-type pro-inflammatory macrophages.

## 4 Discussion

Diabetes mellitus, as a kind of well-known chronic disease worldwide, seriously threatens patients’ health and quality of life, thereby severely restricting the economic and social development of the country [26]. Currently, diabetes mellitus can be classified into three types according to its pathogenesis. The first type is T1DM, mainly characterized by β-cell failure; the second type is T2DM, primarily distinguished by β-cell dysfunction and/or insulin resistance; and the last one is gestational diabetes [27]. Studies have shown that the highest prevalence among the three types is T2DM, which accounts for approximately more than 90% of patients with diabetes [28]. Therefore, there are much more studies related to T2DM. Our study found that miR-335-3p can play a regulatory role in the polarization of macrophages, and it can regulate cell polarization through the target gene IGF-1, thus affecting the process of T2DM.

Several studies have shown that low-grade chronic inflammation and increased macrophages have been identified as important events in the development of obesity and T2DM [29]. Studies showed that only macrophage infiltration occurred in the adipose tissue of obese people, while it did not occur in normal people. This phenomenon is linked to insulin resistance [30].

T2DM development is greatly influenced by macrophage polarization. When LPS activates macrophages, they release TNF-α and iNOS, while interleukin-2 activates macrophages into the M2 state (IL-4). It mainly expresses cellular markers like arginase 1 and IL-10 [31]. It has been shown that when macrophages shift to the M1 phenotype, macrophages secrete pro-inflammatory factors so that the body is in a hypo-inflammation state, which ultimately leads to insulin resistance [32].

IGF-1, as an important member of the system of insulin-like growth factors, is highly homologous to insulin. IGF-1 in the blood is mainly secreted by the liver. It enters the bloodstream, binds to IGF-1 combining proteins in the blood, and is then transported to target organs such as muscle and bone to exert its biological potency. It is a multifunctional class of cellular value-added regulatory factors. Since the 1970s, numerous studies have been conducted on the immune abnormalities in various animal models of autoimmune diabetes. Relevant studies have shown that IGF-1 not only reduces both serum insulin levels and normal blood glucose levels but also improves insulin resistance in patients with T2DM and worse severe insulin resistance. Small clinical trials have demonstrated the potential value of rhIGF-1 in a small subset of patients with severe insulin resistance, where the risk–benefit ratio appears to favor using the drug to address biochemical abnormalities and clinical symptoms [33].

Numerous studies have demonstrated that various miRNAs have important regulatory roles in the course of T2DM. Researchers have found that miRNAs are significantly characterized as being in a dysregulated state in diabetic patients. Therefore, miRNAs can be used as clinical markers for the treatment of T2DM [34]. Meanwhile, related studies have indicated that miRNAs have an effect on the course of T2DM by the regulation of macrophage polarization [35]. miR-375, specifically expressed in pancreatic islet tissues, is one of the most studied diabetes-related miRNAs. The miR-375 overexpression in β cells inhibits glucose-induced insulin secretion. In addition, it has been discovered that miR-375 can limit insulin release by adversely regulating the expression of IGF-1[36]. miR-335-5p inhibited the proliferation of glucose-treated pancreatic cells and promoted apoptosis, which subsequently aggravates T2DM, by inhibiting SLC2A4 expression [37]. miR-335-5p regulates VASH1/TGF-β. Signaling pathways induce insulin resistance and pancreatic islets in gestational diabetic mice β-cell secretion [38].

To demonstrate that a high-fat diet may cause an imbalance in glucose tolerance, this study first created a high-fat diet-induced T2DM animal model. Second, it suggested that the miR-335-3p’s levels were decreased in adipose tissue macrophages of the T2DM mouse model. This indicates that the miR-335-3p’s levels are adversely correlated with macrophages in the T2DM mouse model. The miR-335-3p mimic was injected intraperitoneally into T2DM mice; analysis was done on the body weight and blood glucose levels. Moreover, the findings showed that the miR-335-3p declined insulin resistance. This suggests that miR-335-3p affects the pathogenesis of T2DM. In addition, studies on M1/M2 polarization showed that in adipose tissue of the T2DM mouse model, miR-335-3p restrained M1 polarization and enhanced M2 polarization. The miR-335-3p’s direct target was then discovered to be IGF-1. Finally, according to the previous study, IGF-1 overexpression increased the expression of iNOS as well as TNF-α in miR-335-3p transfected macrophages in a T2DM mouse model, while down-regulating the IL-10 and Agr-1 expression. It was shown that high IGF-1 expression might enhance the conversion of LPS-induced macrophages to M1 pro-inflammatory macrophages as well as inhibit IL-4-induced conversion of macrophages to M2-type anti-inflammatory macrophages. Thus, these results confirm that miR-335-3p targets IGF-1 to modulate macrophage polarization homeostasis, and the miR-335-3p levels are adversely linked with IGF-1 levels. There are some limitations in this study, which only uses animal models and lacks the support of clinical trials and data. The results of the study provide new research directions for the prevention and treatment of T2DM, but translating these findings into clinical applications still needs more clinical trials and further validation in practice.

In summary, miR-335-3p can play a regulating role in macrophage polarization. It can regulate macrophage polarization through the target gene IGF-1, which in turn affects the T2DM process. This study might suggest a new research direction for T2DM prevention and treatment.
